# Evolutionary Trends of Players’ Technical Characteristics in the UEFA Champions League

**DOI:** 10.3389/fpsyg.2020.01032

**Published:** 2020-06-16

**Authors:** Qing Yi, Hongyou Liu, George P. Nassis, Miguel-Ángel Gómez

**Affiliations:** ^1^School of Physical Education and Sport Training, Shanghai University of Sport, Shanghai, China; ^2^Shanghai Key Lab of Human Performance, Shanghai University of Sport, Shanghai, China; ^3^Key Laboratory of Diagnosis & Analysis of Skills & Tactics in Sports, Shanghai University of Sport, Shanghai, China; ^4^School of Physical Education & Sports Science, South China Normal University, Guangzhou, China; ^5^Department of Sports Science and Clinical Biomechanics, Faculty of Health Sciences, SDU Sport and Health Sciences Cluster, University of Southern Denmark, Odense, Denmark; ^6^Facultad de Ciencias de la Actividad Física y del Deporte (INEF), Universidad Politécnica de Madrid, Madrid, Spain

**Keywords:** football, soccer, performance analysis, technical evolution, UEFA Champions League

## Abstract

The current study aimed to investigate the evolutionary trends of players’ technical performances in the UEFA Champions League. Match statistics of 18 technical performance indicators from 1,125 matches (2,489 players, 16,247 full match observations) from the group and knockout stages of the UEFA Champions League (season 2009/2010 to 2017/2018) were analysed. Separate Poisson regression models were run in the generalised mixed linear modelling to compare the differences in technical performances among seasons, and the autocorrelation function was used to identify the correlations within technical variables. Results demonstrated that players’ match performances in variables of shots and shots on target showed trivial changes over the nine seasons. The defending related variables showed either downward trends or negligible changes, and the passing- and attacking-related variables showed different evolving paths throughout the course of the nine seasons. These findings may indicate that European teams are now more focussed on the control of match play, creating offensive space by increasing passing frequency and accuracy rather than crossing the ball from the wings into the penalty box. The significant autocorrelations were only detected in the attacking- and passing-related variables of crossing, though ball and aerial wins, they displayed persistence patterns among the nine seasons.

## Introduction

Football matches are characterised by high dynamicity (Garganta, 2009) where the players’ adapt their behaviours in the continuous performer–environment interactions (Barreira et al., 2015; Aquino et al., 2017b). Thus, the variability of players’ match performances can be observed and assessed between matches (Bush et al., 2015a; Liu et al., 2016a). The within-season variation was observed at a low level (Morgans et al., 2014), but when putting it in a larger timeframe, the playing patterns of football matches have undergone substantial changes over the past seasons and will continue to evolve due to the interaction of external factors (Bush et al., 2015b) and intrinsic variation within the human movement (Gonçalves et al., 2014). The modification of playing rules, the innovation of tactics, and the advancement of technical and physical preparation have been reported as the main external contributors of the longitudinal performance changes (Barnes et al., 2014; Wallace and Norton, 2014). Nevertheless, in addition to the causes of performance evolution, it is also important to figure out how the playing patterns evolve during a given period. The patterns of match play can be described by a selection of match actions and events that could be valid measurements of various aspects of match performance (O’Donoghue, 2009; McGarry et al., 2013). Naturally, tracking the longitudinal changes of performance parameters may provide a valid way to interpret and quantify the evolution of playing patterns. Furthermore, technical parameters have been identified to be related to the match outcome (Castellano et al., 2012; Liu et al., 2016b). Therefore, the investigation of the evolutionary trends of technical parameters allows the understanding of how coaches find the secret to succeed in a match.

To date, literature about the evolution of technical parameters is well documented. Williams et al. (1999) reported that players from the top tier of English football performed more passes, dribbles, and crosses during the period of season 1991/1992 and 1997/1998. Subsequent research from Barnes et al. (2014) in the English Premier League found that the total number of passes made by players per match increased by 40% and the number of short and medium passes increased, but long passes varied little across the timeframe of the study (season 2006/2007–2012/2013). Furthermore, more detailed research has also been conducted that attempts to identify the long-term trends of the technical characteristics in European domestic leagues considering the effects of playing positions (Bush et al., 2015b), team quality (Bradley et al., 2016), player identity (Bush et al., 2017), and match outcome (Konefał et al., 2019). However, each national league was characterised by different specificities and behaviours of match play (Dellal et al., 2011; Oberstone, 2011). Thus, the identified evolutionary dynamics of the match performance in a specific domestic league cannot represent all evolving trends of match play in modern football. A previous study Wallace and Norton (2014) analysed the evolution of match play in an international competition (FIFA World Cup) over a 44 year period. The research focussed on the longitudinal changes of the game structure, speed, and play patterns rather than the evolution of technical parameters.

However, limited technical performance parameters were analysed in the abovementioned literature, and the studies were mainly focussed on passing related parameters. These findings may provide limited information of the overall evolutionary process of players and teams’ performance. Furthermore, in addition to the comparison of technical performance among seasons, the evolving trends of match play could also be identified by the measure of the correlations of technical match actions and events among seasons (Yi et al., 2019a). The autocorrelation function (ACF) has been reported as a valid measure (temporal series) to assess the dynamic correlations among a time series (Prieto et al., 2016). This dynamic time-dependent approach may provide a novel insight to assess the temporal relationships within technical variables in a specific period of time.

The UEFA Champions League is considered to be one of the top international competitions and the participating teams are top squads from all over Europe, leading the latest trends in modern football (Liu et al., 2015). Coaches and performance analysts from these teams have been devoted to the innovation of tactics and strategies in order to improve the players’ match performance (Memmert and Rein, 2018). The results from the previous studies revealed the temporal changes over a specific period, but the playing patterns of football matches evolve over time; more recent changes need to be identified based on the latest database to describe the contemporary trends of the football match. Therefore, the current study aims to explore the evolutionary trends of players’ technical parameters in the UEFA Champions League from season 2009/2010 to 2017/2018 and provide a comprehensive understanding of how the technical characteristics evolve in modern football incorporating the match performance of players from different European countries in goal scoring, attacking, passing, and defending aspects.

## Materials and Methods

### Data Source and Reliability

Technical match performance data of players in the UEFA Champions League across nine consecutive seasons (2009/2010–2017/2018) were acquired from a public-accessed football statistic website called “whoscored.com,^1^” whose data have been considered highly reliable and were used in previous studies (Liu et al., 2015; Yi et al., 2019b), as the data provider is the OPTA Sports. The inter-operator reliability of the tracking system (OPTA Client System) has been previously verified (*Kappa* values > 0.90) with high consistency when repeatedly coding the match actions and events (Liu et al., 2013). The study design and procedures were in accordance with the Declaration of Helsinki and approved by the ethics committee of the local university.

### Sample and Technical Parameters

The sample of this study comprises the technical match statistics of 125 matches per season (total matches analysed = 1,125 matches; 2,489 players; and 16,247 players’ observations) in the group stage and knockout stage of the UEFA Champions League from season 2009/2010 to 2017/2018 (*n* = 1,808; *n* = 1,832; *n* = 1,796; *n* = 1,800; *n* = 1,793; *n* = 1,796; *n* = 1,809; *n* = 1,803; and *n* = 1,810 players’ observations, respectively). Only the outfield players that played at least one full match were included for further analysis to make sure that match observations could be analysed upon the same time dimension. Eighteen technical performance-related actions and events were analysed and classified into three groups of variables (goal scoring, attacking and passing, and defending) in the analysis referring to the previous studies (Lago-Peñas et al., 2010; Liu et al., 2015; Yi et al., 2019b). The grouping information and operational definitions of these technical variables are in Table 1.

**TABLE 1:** Selected technical performance-related match events and actions.

**Groups**	**Event or action: operational definition**
Variables related to goal scoring	Shot: an attempt to score a goal, made with any (legal) part of the body, either on or off target
	Shot on target: an attempt to goal which required intervention to stop it going in or resulted in a goal/shot which would go in without being diverted
Variables related to passing and organising	Touch: a sum of count values of all actions and events where a player touches the ball
	Pass: an intentional played ball from one player to another
	Pass accuracy (%): successful passes as a proportion of total passes
	Key pass: the final pass or cross leading to the recipient of the ball having an attempt at goal without scoring
	Cross: any ball sent into the opposition team’s area from a wide position
	Long ball: an attempted pass of 25 yards or more
	Through ball: a pass that split the last line of defense and plays the teammate through on goal
	Dribble: a dribble is an attempt by a player to beat an opponent in possession of the ball. OPTA also log attempted dribbles where the player overruns the ball
	Aerial won: two players competing for a ball in the air, for it to be an aerial duel both players must jump and challenge each other in the air and have both feet off the ground. The player who wins the duel gets the *Aerial won*, and the player who does not gets an *Aerial lost*
	Fouled: where a player is fouled by an opponent
	Offside: awarded to the player deemed to be in an offside position where a free kick is awarded. If two or more players are in an offside position when the pass is played, the player considered to be most active and trying to play the ball is given offside.
Variables related to defending	Tackle: the action of gaining possession from an opposition player who is in possession of the ball
	Interception: a player intercepts a pass with some movement or reading of the play
	Clearance: attempt made by a player to get the ball out of the danger zone, when there is pressure (from opponents) on him to clear the ball
	Foul: any infringement that is penalised as foul play by a referee
	Yellow card: where a player was shown a yellow card by the referee for reasons of foul, persistent infringement, hand ball, dangerous play, etc.

### Statistical Analysis

Separate Poisson regressions were run in the generalised mixed linear modelling performing with *Proc Glimmix* in the University Edition of Statistical Analysis System (version SAS Studio 3.6) used to examine both the differences in technical variables between seasons and the localised differences verified. The value of each of the 18 technical performance-related variables was selected as the dependent variable (Yi et al., 2019c). The fixed effects estimated the effects of match location (home, away, and neutral), competition stage (group stage and knock-out stage), match outcome (win, draw, and loss) and playing position (central defender, full back, central midfielder, wide midfielder, and forward), as well as the team and opponent strength estimated by including the difference in the log of the end-of-season UEFA club coefficient as a predictor (Yi et al., 2018). The player identity was employed as the random effect to account for the repeated-measure data acquired from players in multiple matches across seasons.

Autocorrelation function was employed to quantify the correlations of a technical variable among a time series of nine seasons with its own values (Yi et al., 2019a). The statistical software IBM SPSS version 25 for Windows (IBM Corp., Armonk, NY, United States) was used for the analysis. The ACF was calculated with a lag length of one season, and seven lags were chosen according to the length of the time series. There was no time-offset if lag = 0. The magnitude of the absolute value of ACF was assessed qualitatively with the following scales: <0.1 trivial, 0.1–0.3 small, 0.3–0.5 moderate, 0.5–0.7 large, 0.7–0.9 very large, >0.9 nearly perfect (Hopkins, 2002). Statistical significance was set at *P* ≤ 0.5.

Uncertainty in the true effects of the predictors was evaluated by a combination of null hypothesis significance testing (*P*-value) and non-clinical magnitude-based inference. An implemented spreadsheet accompanying the package of materials for generalised mixed modelling with SAS Studio was used for the evaluation (Hopkins, 2016). The magnitude of meaningful difference and the 90% confidence limit were expressed in standardised units, and a standardised effect of 0.2 and −0.2 were assumed to be the smallest worthwhile differences. Effect were considered clear if the 90% confidence limit of the effect size did not affect the smallest worthwhile differences of 0.2 and −0.2 simultaneously. Estimated magnitudes of effect sizes were quantified by the following scales: <0.2 trivial, 0.2–0.6 small, 0.6–1.2 moderate, and 1.2–2.0 large (Hopkins et al., 2009), along with a qualitative likelihood of the clear effects: <0.5%, most unlikely; 0.5–5%, very unlikely; 5–25%, unlikely; 25–75%, possibly; 75–95%, likely; 95–99.5%, very likely; and >99.5%, most likely (Hopkins et al., 2009).

## Results

### Goal Scoring Related Variables

**FIGURE 1:**
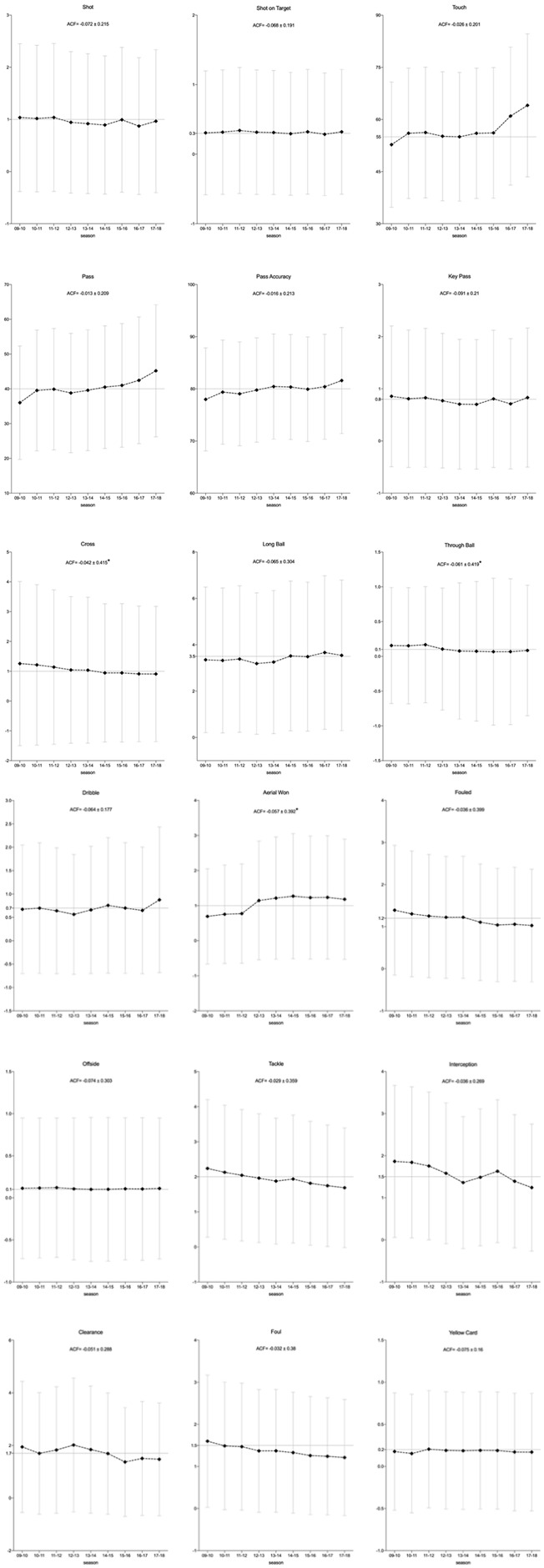
Temporal changes of each technical performance indicators from season 2009/2010 to 2017/2018. Black dot and error bar indicate the mean value and standard deviation, respectively. Value of ACF is presented as mean value of 7 lags with the form of mean ± standard deviation. Asterisk indicate the statistical significance.

The number of shots and shots on target showed trivial differences across nine seasons (*p* < 0.9685; effect size (ES) = −0.16, 0.1; likelihood: likely-most likely); shots on target observed a relatively greater fluctuation (see Figure 1). There were no clear correlations for these variables over the same period of time (ACF = −0.072 ± 0.215, −0.068 ± 0.191; *P* = 0.52, 0.575) (see Table 2).

**TABLE 2:** Descriptive statistics of technical match performance of players in the UEFA Champions League from season 2009/10 to 2017/18.

**Variable**	**Season**									**Total (*n* = 16,247)**
	**2009–2010 (*n* = 1,808)**	**2010–2011 (*n* = 1,832)**	**2011–2012 (*n* = 1,796)**	**2012–2013 (*n* = 1,800)**	**2013–2014 (*n* = 1,793)**	**2014–2015 (*n* = 1,796)**	**2015–2016 (*n* = 1,809)**	**2016–2017 (*n* = 1,803)**	**2017–2018 (*n* = 1,810)**	
Shot	1.041.42	1.021.41	1.041.42	0.941.36	0.921.34	0.891.32	0.991.39	0.871.31	0.971.37	1.151.54
Shot on Target	0.310.89	0.320.89	0.340.91	0.320.89	0.310.89	0.290.88	0.320.90	0.290.88	0.320.90	0.400.79
Touch	52.7618.01	56.0318.76	56.2518.81	55.1918.57	55.0218.53	56.0218.75	56.1318.78	60.9719.87	64.0620.56	62.1522.18
Pass	36.0216.32	39.5417.36	39.9017.47	38.8017.14	39.5917.38	40.5017.64	40.9917.79	42.4518.21	45.2019.01	46.2721.41
Pass Accuracy (%)	77.989.87	79.379.99	79.049.96	79.7810.02	80.4410.07	80.3510.06	79.9410.03	80.4110.07	81.5910.16	81.5010.28
Key Pass	0.861.35	0.811.32	0.831.33	0.771.29	0.711.24	0.701.24	0.811.32	0.711.25	0.831.33	0.891.22
Cross	1.262.75	1.212.69	1.142.59	1.042.46	1.042.45	0.942.32	0.952.32	0.912.28	0.912.27	1.762.59
Long Ball	3.353.14	3.323.13	3.383.16	3.193.05	3.253.09	3.523.23	3.493.21	3.673.31	3.543.25	4.583.78
Through Ball	0.160.83	0.150.83	0.170.84	0.110.88	0.080.98	0.071.00	0.071.06	0.071.05	0.080.94	0.220.62
Dribble	0.671.38	0.701.40	0.641.35	0.561.28	0.661.36	0.761.45	0.701.40	0.651.35	0.881.56	0.851.34
Aerial Won	0.691.36	0.751.40	0.771.42	1.151.69	1.221.74	1.271.78	1.231.75	1.241.76	1.181.72	1.181.50
Fouled	1.391.54	1.301.49	1.251.46	1.221.45	1.221.45	1.111.38	1.041.35	1.061.36	1.031.34	1.161.27
Offside	0.110.84	0.120.83	0.120.83	0.110.84	0.100.86	0.100.85	0.110.84	0.110.85	0.110.84	0.200.58
Tackle	2.241.96	2.131.91	2.051.87	1.961.84	1.881.80	1.941.82	1.821.77	1.751.73	1.691.71	2.071.78
Interception	1.861.81	1.841.79	1.761.76	1.581.67	1.361.57	1.491.63	1.631.70	1.391.58	1.241.51	1.891.72
Clearance	1.942.49	1.702.31	1.832.41	2.022.55	1.842.42	1.692.31	1.372.07	1.502.17	1.472.14	2.673.04
Foul	1.601.57	1.491.52	1.471.51	1.371.46	1.371.46	1.331.44	1.261.40	1.241.39	1.211.38	1.271.26
Yellow Card	0.180.70	0.150.70	0.200.70	0.190.70	0.190.70	0.190.70	0.190.70	0.170.70	0.170.70	0.180.38

**n* denotes the number of players’ observations. Results are presented as the form of mean ± standard deviation, representing the average value that players achieved in a full match. Units are counts, except for pass accuracy. The average value was calculated dividing the total number of actions by the number of players’ observations.*

### Attacking and Passing Related Variables

Similar changing trends were observed among touches, passes, and pass accuracy (%) during the timeframe of this study. There were simultaneous increases between season 2009/2010 and 2010/2011 (52.76 ± 18.01vs. 56.03 ± 18.76, 36.02 ± 16.32 vs. 39.54 ± 17.36, 77.96 ± 9.87 vs. 79.37 ± 9.99; *P* < 0.0001; ES = 0.18, 0.21, 0.14; likelihood: possibly, possibly, and very likely), which then remained relatively steady until season 2016/2017, where the significant increases were appealed again peaking at season 2017/2018 (64.06 ± 20.56, 45.20 ± 19.01, 81.59 ± 10.16), although trivial increases for pass and pass accuracy were observed between season 2015–2016 and 2016–2017 (40.99 ± 17.79 vs. 42.45 ± 18.21, 79.74 ± 10.03 vs. 80.41 ± 10.07; *P* = 0.0033 and 0.147; ES = 0.08, 0.05; likelihood: most likely and most likely). The players’ match performances in crosses and fouls presented downward trends over the nine seasons. Even though the differences among most of the seasons were trivial, the number of crosses and fouls of players obtained per match decreased from 1.26 ± 2.75 and 1.39 ± 1.54 in 2009/2010 to 0.91 ± 2.27 and 1.03 ± 1.34 in 2017/2018 (*P* < 0.0001; ES = −0.21, −0.32; likelihood: possibly, most likely). The longitudinal changes in through ball and aerial wins showed opposite trends; there was a pronounced decrease for through ball and an increase for aerial wins in season 2012/2013 compared to season 2011/2012 (0.17 ± 0.84 vs. 0.11 ± 0.88, 0.77 ± 1.42 vs. 1.15 ± 1.69; *P* < 0.0001; ES = −0.20, 0.29; likelihood: possibly, most likely). Afterwards, both showed negligible changes over following seasons. The number of dribbles varied little within season 2009/2010 and 2016/2017, while it significantly increased by 35% between season 2016/2017 and 2017/2018 (0.65 ± 1.35 vs. 0.88 ± 1.56; *P* < 0.0001; ES = 0.20; likelihood: possibly). Players’ match performances in key passes, long balls, and offsides varied by trivial magnitudes across the nine seasons (*P* < 0.9916; ES = −0.16, 0.16; likelihood: likely-most likely).

Concerning the inter-variable correlations among the nine seasons, the statistical significance was only observed in cross, through ball, and aerial wins (ACF = −0.042 ± 0.415, −0.061 ± 0.419, −0.057 ± 0.392; *P* = 0.036, 0.021, 0.033) showing trivial negative correlations among seasons.

### Defending Related Variables

Continued momentum of decline can be found in the number of tackles and fouls of players performed per match, declining from 2.24 ± 1.96, 1.60 ± 1.57 in season 2009/2010 to 1.69 ± 1.71, 1.21 ± 1.38 in season 2017/2018 (*P* < 0.0001; ES = −0.36, −0.32; likelihood: most likely, most likely). Although there were also general declining trends in interception and clearance, they experienced fluctuations during the nine seasons. Players achieved more interceptions and clearances (*P* < 0.0001; ES: −0.47, −0.26; likelihood: most likely, likely) in season 2009/2010 (1.86 ± 1.81; 1.94 ± 2.49) compared to season 2017/2018 (1.24 ± 1.51; 1.47 ± 2.14), peaking at season 2009/2010 (1.86 ± 1.81) and 2012/2013 (2.02 ± 2.55), respectively. Players showed a relatively stable match performance in yellow cards among these nine seasons (*P* < 0.9875; ES = −0.10, −0.13; likelihood: very likely-most likely). All defending related variables showed non-clear correlations during the studied period of time (ACF = −0.075 ± 0.16 to −0.029 ± 0.359; *P* = 0.079–0.784).

## Discussion

The current study quantified the long-term trends of technical performance indicators among seasons aiming to identify the contemporary evolution of players’ technical characteristics based on the latest nine seasons of the UEFA Champions League. The evolutional process of 18 technical performance related indicators regarding goal scoring, attacking and passing, and defending aspects have been demonstrated. The effects of situational factors and positional roles were controlled by the modelling to deal with the intrinsic variation within matches.

Coaches and performance analysts have been trying to find ways to increase the scoring opportunities and improve the efficiency over years, so the increase in relevant match statistics, therefore, might be expected. However, our research on the players from the UEFA Champions League demonstrated that their match performances in variables of shots and shots on target showed trivial changes over the last nine seasons. This finding is in line with a study on the English Premier League from Barnes et al. (2014) in which the evolutionary trends of players’ technical performance were identified based on a period of seven consecutive seasons. Nevertheless, opposite changes were observed in Germany’s Bundesliga, the number of total shots decreased among playing positions during season 2014/2015 to 2016/2017 (Konefał et al., 2019). This disparity may be due to the relatively smaller database used (three seasons); the identified longitudinal performance characteristics cannot be compared with the seven-season and nine-season period studies. The results among studies may be more consistent by expanding the timeframe of the study on the German Bundesliga.

Although the number of shots and shots on target varied little, the number of touches and passes players performed per match increased over the last nine seasons. This trend partly contrasts with the research by Tenga et al. (2010) who argued that longer passing sequences could produce more shots per possession. This may indicate that the increase in the frequency of passes cannot directly bring more scoring opportunities, the ability of creating offensive space and sending the ball into the scoring area may play an important role (Collet, 2013). This statement could be supported by the changes of key pass and through balls as these two match performance indicators can describe the key situations during match play. The evolving technical characteristics of the increase of passing frequency may possibly be driven by the prevalence of possession play (Aquino et al., 2017a; Yi et al., 2019b), whereas how to decrease unwanted passes and improve offensive efficiency are key issues for teams that employ this tactical approach to consider during the coaching process (Alves et al., 2019). These evolving trends are further supported by the changes in the number of crosses and long balls. These variables showed a relentless decline and a limited fluctuation, respectively, among nine seasons, which means that players performed more short passes and medium passes, so the pass accuracy was increased accordingly. Similar findings were reported in a previous study on the English Premier League (Barnes et al., 2014). This indicates that the passing tempo increased during the period of nine seasons and teams tended to play a more elaborate match.

The changes of passing actions may also influence players’ performance in attacking behaviours. The players’ stable performance in offside could be considered as a result of the minimal changes of the key pass and through balls over the same period, as these two technical performance indicators are usually associated with the offside situations. The number of key passes fluctuated within a narrow range, and the mean number of through balls players achieved per match remained at a relatively low level, although there was a clear decrease in season 2012/2013. Combining this result with the feature of the occurrence rate of offsides during a match may explain the low variation of offsides over the nine seasons. Another concern is that the number of crosses continuously decreased and the number of long balls slightly changed among the period of nine seasons, while the percentage of the aerial wins observed a significant increase, especially from the season 2012/2013. This result is in line with the study of Konefał et al. (2019) on the German Bundesliga. As the aerial duels usually occurred from crosses and long balls (Liu et al., 2016a), our findings may indicate that the decrease of the crosses has a negative impact on the appearance of the aerial duels. However, this finding should be interpreted with caution, because the underlying causes need to be verified and more insight is needed to interpret the substantial increase in the last six seasons.

The occurrence frequency of dribbling was relatively stable in the first eight seasons and then suddenly increased in the last season. This finding is not in accordance with the research made by Williams et al. (1999), who reported that the incidence of dribbling in the English league increased from season 1991/1992 to 1997/1998. Future research is needed given that dribbling is an important performance indicator explaining players’ technical characteristics and tactical roles during match play. Another interesting finding of our analysis is that the number of players who were fouled during a match showed a steady decline during the timeframe of the current study. Together with the findings in defensive performance indicators, the number of tackles and fouls also showed a similar downward trend and the number of yellow cards remained stable among the nine seasons. These findings support the notion of Oberstone (2011) that players played football more cleanly. The modification of rules could be one of the potential reasons for this data. The rules of the game are being tweaked over time as the lawmakers continue to figure out the best way to regulate the behaviour of players on the pitch, especially in controversial and confrontational situations involving opponents or match officials. The downward trends could also be found in the number of interceptions and clearances, even though fluctuations were seen. These findings may reflect the way teams’ defence has changed over time, rather than the retrogress of players’ defensive abilities. The defensive manners of teams are evolving in the direction of joint actions, which can improve the effectiveness of the defence.

Autocorrelation functions of technical variables provide important understanding from another perspective to depict how playing patterns evolve. A prior study investigated the long-term trends of the variation of technical variables in the UEFA Champions League and reported that the variation of through balls displayed trivial negative autocorrelation throughout a time period of eight seasons (2009/2010–2016/2017) (Yi et al., 2019a). This finding was also identified in the current study, where crosses, through balls, and aerial wins showed substantial negative correlations among nine seasons, while the temporal relationships within other variables have not been detected. The lower ACF value indicates less persistence among a time series (Prieto et al., 2017). However, the magnitudes of the autocorrelations were trivial, which may still provide useful insights about the patterns of these three variables to describe the dynamic trends of technical performances. These findings may mean that the more unstable the performance of the players in the form of crosses, through balls, and aerial duels won in a season, the more stable the performance could be of these variables in the following season. Thus, these susceptible variables should be treated by coaches with care.

## Conclusion

The current study investigated the evolving patterns of players’ technical performance indicators based on a dataset of nine seasons from the UEFA Champions League. Disparities exist in the evolutionary patterns between technical performance indicators of goal scoring, passing, organising, and defending. Players performed an increasing number of passes per match, especially short passes, with no change in the number of shots and shots on target. Teams are now more focussed on controlling the match and creating offensive space by increasing the passing frequency and accuracy, rather than crossing the ball from the wings into the penalty box. However, the progression of the ability of creating scoring opportunities was not observed and the success rate of aerial duel increased. We also found that all defending related indicators did not show upward trends during the nine seasons. The number of players who were fouled decreased during the match and teams organised their defence collectively in a group-tactical way to enhance the effectiveness of defensive actions. Besides, only the attacking and passing related variables of crosses, through balls, and aerial wins demonstrated a certain degree of persistence over the nine seasons.

### Practical Applications

We tracked the temporal trends of the technical match performances of contemporary elite football and the longitudinal changes were quantified across a period of nine seasons. We provided important evidence to explain how and why the technical aspect of match-play evolve. The identified evolving characteristics could provide a holistic understanding for coaches and performance analysts to fine-tune their performance knowledge about the matches in the UEFA Champions League. The key performance indicators such as shots on target, key passes, and through balls should be treated with care; special interventions are needed to improve players’ match performance towards these indicators. Teams could also diagnose and optimise their tactics and strategies according to the identified trends. For example, the current study reported that the number of passes increased while the number of crosses decreased, which may provide references for teams in the disposition of defensive tactics. However, this study failed to consider the effects of playing positions and situational variables, thus some important information was masked as the differences exist in players’ tactical roles and duties and in the technical behaviours when playing in different situations. Future research regarding the investigation of evolving trends of technical indicators should take players’ positions on the pitch and the competing situations into account, which may contribute to more practical insights. Moreover, further research is needed to explore the causative factors underlying the temporal changes in players’ technical performance. The modification of rules, the improvement of skill execution, and the innovation of tactics may help to explain the dynamic changes.

## Data Availability Statement

All datasets generated for this study are included in the article/supplementary material.

## Ethics Statement

The studies involving human participants were reviewed and approved by Shanghai University of Sport. Written informed consent for participation was not required for this study in accordance with the national legislation and the institutional requirements. Written informed consent was not obtained from the individual(s) for the publication of any potentially identifiable images or data included in this article.

## Author Contributions

QY, M-ÁG, and HL: conceptualisation and methodology. HL: software. QY: data collection, writing – original draft preparation, visualisation, and funding acquisition. HL, GN, and M-ÁG: writing – review and editing and supervision.

## Conflict of Interest

The authors declare that the research was conducted in the absence of any commercial or financial relationships that could be construed as a potential conflict of interest.

## Footnotes

1 https://www.whoscored.com
